# Association between lesion location and sensorimotor rhythms in stroke – a systematic review with narrative synthesis

**DOI:** 10.1007/s10072-023-06982-8

**Published:** 2023-08-22

**Authors:** Ivana Kancheva, Sandra M. A. van der Salm, Nick F. Ramsey, Mariska J. Vansteensel

**Affiliations:** UMC Utrecht Brain Center, Department of Neurology & Neurosurgery, University Medical Center Utrecht, P.O. Box 85060, 3508 AB Utrecht, The Netherlands

**Keywords:** Systematic review, Stroke, Lesion location, Sensorimotor rhythms, Motor recovery, Brain-Computer Interfaces

## Abstract

**Background:**

Stroke causes alterations in the sensorimotor rhythms (SMRs) of the brain. However, little is known about the influence of lesion *location* on the SMRs. Understanding this relationship is relevant for the use of SMRs in assistive and rehabilitative therapies, such as Brain-Computer Interfaces (BCIs)..

**Methods:**

We reviewed current evidence on the association between stroke lesion location and SMRs through systematically searching PubMed and Embase and generated a narrative synthesis of findings.

**Results:**

We included 12 articles reporting on 161 patients. In resting-state studies, cortical and pontine damage were related to an overall decrease in alpha (∼8–12 Hz) and increase in delta (∼1–4 Hz) power. In movement paradigm studies, attenuated alpha and beta (∼15–25 Hz) event-related desynchronization (ERD) was shown in stroke patients during (attempted) paretic hand movement, compared to controls. Stronger reductions in alpha and beta ERD in the ipsilesional, compared to contralesional hemisphere, were observed for cortical lesions. Subcortical stroke was found to affect bilateral ERD and ERS, but results were highly variable.

**Conclusions:**

Findings suggest a link between stroke lesion location and SMR alterations, but heterogeneity across studies and limited lesion location descriptions precluded a meta-analysis.

**Significance:**

Future research would benefit from more uniformly defined outcome measures, homogeneous methodologies, and improved lesion location reporting.

**Supplementary information:**

The online version contains supplementary material available at 10.1007/s10072-023-06982-8.

## Introduction

Stroke is a leading cause of mortality and disability worldwide with over 5.5 million deaths and 80 million survivors annually [1]. One of the most common clinical sequelae of stroke is motor impairment, which affects approximately 80% of patients and leads to difficulties with tasks of everyday life [2]. Different treatment strategies have been used to restore post-stroke motor deficits. Conventional therapeutic approaches typically focus on strength, endurance, and functional practice with the paretic limb, and have resulted in impairment decreases [3]. More intense interventions, such as constraint-induced movement therapy, have yielded better results, generalizing to increased functional independence [4].

In the past decades, Brain-Computer Interfaces (BCIs) emerged as assistive and rehabilitative technologies for individuals with profound motor disability, such as those with locked-in syndrome (LIS) or (severe) paralysis after stroke [5]. A BCI acquires brain signals in real time and extracts specific features from these signals that reflect the user’s intent. These features are then translated to digital control commands, which can operate computer applications, thereby replacing or restoring the brain’s lost natural output. To illustrate, there is increasing evidence that BCIs can be used for communication through brain-controlled typing software in individuals who have lost the ability to speak [6–8] or as a channel for environmental control (e.g., controlling an assistive robotic arm) for people with paralysis [9–12]. In addition, BCIs may provide users with sensory feedback about their movement attempts, through brain-signal-driven functional electrical stimulation of the muscles [13] or micro-stimulation of the somatosensory cortex surface [9].

Of particular relevance as BCI input are the sensorimotor rhythms (SMRs) present in the neuroelectrical responses recorded from the sensorimotor cortex [14]. The SMRs are generated by complex thalamocortical networks, and they are implicated in important motor functions. A prominent characteristic of SMRs is the attenuation of their amplitude during preparation for, and execution of, movement. This phenomenon, termed event-related desynchronization (ERD), occurs during imagined, passive, or active execution of a motor act and is typically detected in the power of the low-frequency band (LFB) alpha/mu (~ 8–12 Hz) component [15]. Similarly, beta rhythms (~ 13–30 Hz) exhibit a decrease prior to and during movement, which is often followed by a short-lasting amplitude enhancement upon its termination, known as event-related synchronization (ERS) or rebound [16]. Movement is also associated with increases in the high-frequency band (HFB; > 30 Hz) power in the sensorimotor cortex, which is most easily measured with implanted electrodes [17].

Previous studies have demonstrated that stroke survivors display abnormalities in the neural activity associated with rest or movement [18–20]. To illustrate, Kaiser et al. (2012) analyzed the ERD/ERS in stroke patients during motor imagery and execution with the paretic (i.e., affected by the stroke) hand [21]. They discovered a weaker ERD in the contralateral (i.e., ipsilesional), compared to the ipsilateral (i.e., contralesional) hemisphere, during motor imagery in patients with a higher degree of impairment. In addition, a more pronounced ERS was revealed on the lesioned side, compared to the non-lesioned side, during motor execution in individuals with greater motor deficits. Similarly, Rossiter et al. (2014) documented reduced beta desynchronization in the ipsilesional primary motor cortex (M1) during a visually guided grip task, which was also related to impairment level [22]. While this evidence suggests that the movement-related brain activity patterns undergo changes following stroke, data are often based on limited numbers of patients with various clinical presentations and heterogeneous brain damage. Hence, it remains unclear if there is a relationship between alterations in sensorimotor cortical activity and the locus of brain injury (lesion location). Understanding such a relationship is relevant for the use of SMRs within BCI settings, where these features are targeted for the generation of reliable control signals. Furthermore, increased knowledge about the role of stroke lesion location on SMR changes may guide the implementation of personalized neurorehabilitation protocols and BCI solutions [23].

Therefore, we systematically examined the literature on the association between lesion location and SMRs in stroke patients.

## Methods

### Protocol registration

This review was registered with the International Prospective Register of Systematic Reviews (PROSPERO) with registration number CRD42021255803 and can be accessed at https://www.crd.york.ac.uk/PROSPERO/display_record.php?ID=CRD42021255803. To enable PROSPERO to focus on COVID-19 registrations during the 2020 pandemic, the registration record of this review was automatically published exactly as submitted. The PROSPERO team has not checked eligibility.

### Search strategy and selection criteria

Studies were identified by a systematic search in PubMed and Embase from inception and were extracted on May 6^th^, 2021, using Mesh terms and relevant keywords (the full electronic search strategy can be found in Supplementary Table 1) following the Preferred Reporting Items for Systematic Reviews and Meta-analyses (PRISMA) statement [24] and its extension PRISMA-S [25]. The completed PRISMA checklists are provided in Supplementary Tables 2 and 3, respectively.

Studies were included if they (1) were articles or articles in press; (2) reported on the association between SMR characteristics and lesion location in at least one individual with stroke; (3) used an electrophysiological technique to measure neural signals; (4) assessed LFB components relevant to motor function (i.e., alpha/mu and/or beta, or any other component alongside alpha/mu and/or beta); (5) included an eyes-open or eyes-closed resting-state or movement-related experimental condition (including active, passive, attempted, or imagined movement); (6) referred to upper limb motor function. Studies were excluded if they were (1) animal studies; (2) other systematic, narrative, or scoping reviews, or meta-analyses; (3) randomized clinical/controlled trials; (4) case reports on conditions other than stroke, (5) of irrelevant type, e.g., surveys, technical notes, conference proceedings, editorials, classifier performance studies; (6) focused on infants, children, or adolescents; (7) written in languages other than English. Importantly, we anticipated that most studies would be based on non-invasive, widely available electrophysiological monitoring systems, such as scalp EEG, which are less reliable in measuring HFB components [26, 27] and thus, decided to limit the scope of our search to LFB changes. Randomized clinical/controlled trials were regarded as ineligible because their study design is best tailored to the assessment of an intervention or treatment in the field of medicine, rather than on examining the influence of a prognostic factor of interest on certain outcome measures. To ensure that no relevant articles were omitted after extracting the search results, email alerts from both used databases were set-up to update the study inclusion and capture all publications until the present date. In addition, a hand-search in Google Scholar was carried out and reference lists of eligible papers were inspected.

One reviewer (I.K.) applied the eligibility criteria by screening titles and abstracts, and their decisions were cross-checked by a second investigator (M.V.). Both raters were involved in the full-text screening.

### Data extraction

The following information was extracted from each study: (1) author(s) and year of publication; (2) study design; (3) study population with number of individuals affected by stroke and controls; (4) participants’ age; (5) participants’ demographics, as relevant to motor function (e.g., handedness and/or level of motor function of upper extremity); (6) stroke phase; (7) lesion location; (8) electrophysiological monitoring method used and number of electrode(s) or sensor(s); (9) location(s) of analyzed electrode(s) or sensor(s); (10) experimental paradigm; (11) outcome(s) of interest; (12) main study finding(s); (13) main study limitation(s). One reviewer (I.K.) extracted the data by means of a customized spreadsheet and a second rater (M.V.) verified them.

### Methodological quality assessment

There is international consensus on how to appraise the quality of prognosis studies in systematic reviews [28]. To evaluate potential sources of bias, here we employed an adapted version of the Quality in Prognosis Studies (QUIPS) tool, because this is a commonly employed, evidence-based instrument with substantial inter-rater agreement. The tool includes questions linked to six bias domains, namely: (1) study participation; (2) study attrition; (3) prognostic factor measurement; (4) outcome measurement; (5) study confounding; (6) statistical analysis and reporting. It is designed so that it can be operationalized for specific study objectives, including specifying key characteristics, adding items if necessary, or omitting irrelevant items. Criteria within each domain are assessed, thereby generating an overall score for each domain as having a ‘low’, ‘moderate’, or ‘high’ risk of bias [29]. We decided to omit the original QUIPS item on study confounding because the studies we included typically focused on their respective outcomes of interest in relation to stroke lesion location as a prognostic factor, without providing a clear definition of confounders or adjusting for their potential influence. Thus, an adapted version of the QUIPS, without items addressing study confounding was used. In addition, we decided to assign ‘high’ risk of bias in the ‘study participation’ category only if stroke samples included fewer than 10 subjects, ‘moderate’ risk of bias for samples between 10 and 20, and ‘low’ risk of bias for samples larger than 20. This decision was based on our recognition of the inherent challenges in conducting research among people with stroke, particularly more severely affected individuals, including the difficulties with recruiting large numbers of subjects who are fit enough to participate, high subject attrition, and the laboriousness of performing electrophysiological assessments on patients.

Two reviewers assessed the quality of the included publications, with respect to the objectives of the current study, independently using an adapted QUIPS Excel spreadsheet (I.K. and M.V.). Each item of the five assessed categories was rated using ‘yes’, ‘partial’, or ‘no’ answers. Afterwards, sources of bias for each domain were evaluated as follows: high quality for studies with low risk of bias, moderate quality for studies with moderate risk, and low quality when there was high risk of bias, respectively. Any discrepancies between raters were resolved in discussion.

### Data synthesis

We used narrative synthesis to summarize the evidence from included studies and present findings in a tabular format. Due to substantial variability in the used lesion location descriptions, frequency band definitions, techniques for measuring brain activity, experimental conditions, and outcome measures across studies, performing a meta-analysis was not feasible.

### Selection of studies

**Fig. 1:**
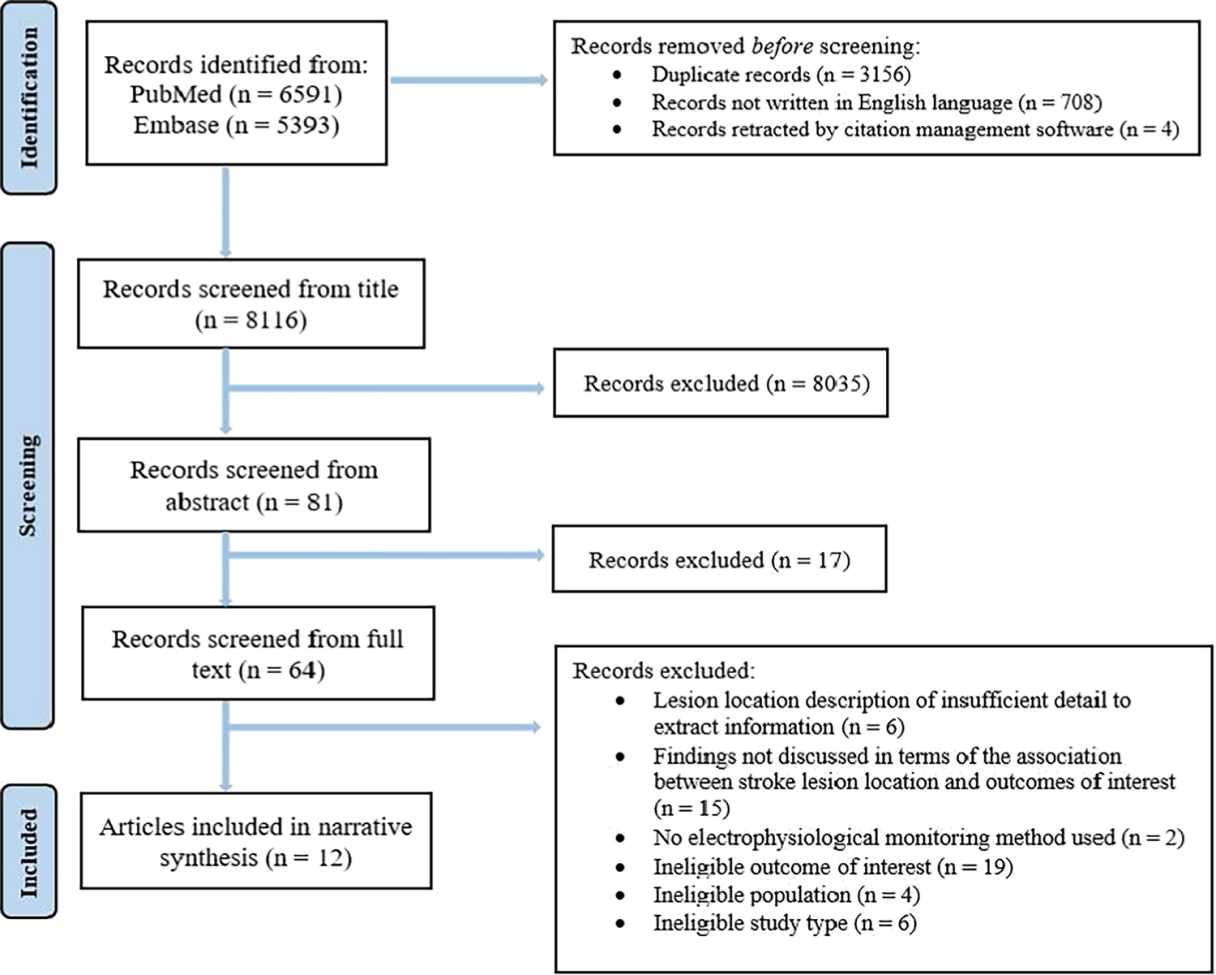
PRISMA flow diagram of study selection. *Note.* Records were deduplicated using the EndNote reference management tool. Titles and abstracts were screened using the Rayyan software [30]. Full-text screening of eligible articles was performed using the Covidence software (Veritas Health Innovation, Melbourne, Australia, www.covidence.org)

A total of 11, 984 publications were screened, of which 64 were considered for full text review, and 12 met the inclusion selection criteria (Fig. 1).

### General study characteristics

The search yielded 8 studies with retrospective case–control designs, 3 retrospective case–control design studies with longitudinal assessments, and 1 retrospective cohort study. Articles reported on 317 participants (161 patients and 156 controls) in total, of which 13 dropped-out or were lost to follow-up in the studies that included longitudinal assessments. The clinical status was acute for 62, subacute for 61, and chronic for 38 patients based on the authors’ descriptions. Stroke type was ischemic for 145, hemorrhagic for 2 (1 intracerebral and 1 pontine), and not specified for 14 subjects with stroke. Regarding the location of the lesions, 49 were cortical, 71 subcortical, 22 mixed (cortico-subcortical), 4 infratentorial (medullar or pontine), and 15 pontine. Four studies included a resting-state and 3 employed a movement attempt/execution paradigm, 4 studies involved both a rest (baseline) and a movement-related task, and 1 involved motor imagery. Concerning participant selection, 6 examinations compared one patient group with the same (cortical or subcortical) lesion location to a control group, 5 studies analyzed sub-groups of patients with different lesion locations against healthy volunteers, and 1 study reported on a single patient’s SMR features in the context of testing a BCI system for control. Table 1 summarizes the characteristics of all included studies.

**Table 1:** General characteristics of included studies (*n* = 12)

Author (year)	Study Design	Population (N subjects)	Age	Handedness and/or Motor Function Score	Stroke Phase	Lesion Location	Electrophysiological Monitoring Method	Analyzed Electrode(s)/Sensor(s)	Experimental Paradigm	Outcome measure(s)	Main Finding(s)	Main Limitation(s)
*Resting-state Studies*												
Van Wijngaarden et al. (2016)	CCr	Ischemic stroke patients (21) and aged-matched controls (17)	Patients: M = 72 (range 38–85) Controls: M = 68 (range 60–80)	Not specified	Acute (M = 69 h after onset, range 21–99 h)	Cortical: MCA territory	19 electrodes EEG	F3, F4, F7, F8, Fz, C3, C4, Cz, P3, P4, Pz, T3, T4, T5, T6, O1 and O2	Resting-state	Mean spectral energy in δ (1–4 Hz), θ (4.2–7.9 Hz), α (8.3–11.9 Hz) and β (12.4–30.6 Hz)	Downward shift of dominant α peaks in EEG power spectra with slowing into a lower frequency in patients (M ± SD = 7.9 ± 0.51 Hz) vs. controls (M ± SD = 9.7 ± 0.56 Hz). Increase in spectral power within δ and θ in patients, compared to controls, over DAM-H F8, C4, T4 and T6 and CON-H T3, O1, O2. Attenuated β power across all electrodes	RD; selection bias; lesion descriptions not detailed. High heterogeneity in the topographical distribution of individual differences in spectral power in the combined frequency range of δ and θ (1–8 Hz). Despite these individual differences, global changes, such as the shift in α peaks, are observed across all patients
Finnigan et al. (2016)^†^	CCr	Ischemic stroke patients (17) and age-matched controls (28)	Patients (M ± SD): 69.3 ± 9.91 (range 51–86) Controls: 70.4 ± 8.58 (range 56–84)	NIHSS (M ± SD): 14 ± 6.32 (range 4–24)	Acute (within 24 hours after onset)	Cortical: MCA territory	19 electrodes qEEG	Fp1, Fp2, F7, F3, Fz, F4, F8, T3, C3, Cz, C4, T4, T5, P3, Pz, P4, T6, O1, O2 (EEG cap or individual electrodes were used)	Eyes-closed resting-state	Relative qEEG bandpower in δ (0.98–3.91 Hz), θ (4.39–7.32 Hz), α (7.81–12.21 Hz), β (12.70–29.79 Hz). DAR, DTABR, and Qslowing* (ratio of summed power across 1.95–7.81 Hz versus summed power across 1.95–24.90 Hz) *Higher DAR, DTABR, and Qslowing scores indicate a greater degree of EEG shift towards low-frequency activity	Higher relative δ power (M = 0.58) in patients vs. controls (M = 0.29). Higher relative θ power (M = 0.17) in patients vs. controls (M = 0.11). Lower relative α power (M = 0.13) in patients vs. controls (M = 0.34). Lower relative β power (M = 0.12) in patients vs. controls (M = 0.26). Higher DAR ratio (M = 6.64) in patients vs. controls (M = 1.34). Higher DTABR ratio (M = 4.25) in patients vs. controls (M = 0.84). Higher Qslowing (M = 0.66) in patients vs. controls (M = 0.36)* *Reported findings are based on analysis of all electrodes	RD; selection bias; lesion descriptions not detailed; possibility that one or a few patients had higher DAR scores (above 3.7) prior to the study due to alpha slowing or cognitive impairment linked to cerebrovascular issues
Fanciullacci et al. (2017)	CCr	Ischemic stroke patients (30) and age-matched controls (10)	Patients (M ± SD): 69.5 ± 13.6 Cortico-subcortical group: 72.2 ± 10.9 Subcortical group: 68.92 ± 10 Controls: 62 ± 10.3	Right-handed; NIHSS cortico-subcortical group: M = 4.9 NIHSS Sub-cortical group: M = 3.8	Subacute (between 10 and 45 days after onset)	Cortico-subcortical: subcortical + cortical involvement (n = 15) Subcortical: deep WM inferior to corpus callosum, IC, thalamus, BG (n = 15)	64 electrodes qEEG	Fp1, Af3, Af7, F1, F3, F5, F7, Fp2, Af4, Af8, F2, F4, F6, F8, Fc1, Fc3, Fc5, Ft7, C1, C3, C5, T3, Cp1, Cp3, Cp5, Fc2, Fc4, Fc6, Ft8, C2, C4, C6, T4, Cp2, Cp4, Cp6, P1, P3, P5, T5, Po7, Po3, O1, P2, P4, P6, T6, Po8, Po4, O2	Eyes-closed resting-state	PSD in DAM-H and CON-H in δ (1–4 Hz) and α (8.1–14 Hz); DAR	**Whole scalp patients:** Higher δ power compared to controls **DAR controls:** 2.02 **DAR Cortico-subcortical group:** 5.16 **DAR Subcortical group:** 2.49 **Cortico-subcortical group:** Higher DAR vs. controls and vs. subcortical group in whole scalp and in CON-H frontal and posterior areas. Higher δ compared to subcortical group in frontal area of CON-H. Higher δ in DAM-H than CON-H δ in whole H and frontal area. Lower α power compared to subcortical group in whole scalp, each H, and each area (frontal, central, posterior in both CON-H and DAM-H) **Subcortical group:** Lower δ power in CON-H vs. DAM-H. Higher α power compared to cortico-subcortical group. Lower DAR in whole scalp and CON-H frontal and posterior areas compared to cortico-subcortical group	RD; selection bias; relatively wide window of enrollment (10–45 days post-stroke)
Babiloni et al. (2010)	CCr	Stroke patients (13*) and age-matched controls (15) *12 infarctions and 1 hemorrhage	Patients (M ± SEM): 51 ± 4.1 Controls: 50.3 ± 1.6	LIS diagnosis: 2 total, 11 classic, as per Bauer classification	Chronic (M = 9.5 months from diagnosis to EEG recording)	Ventral pons	19 electrodesEEG	Fp1, Fp2, F7, F3, Fz, F4, F8, T3, C3, Cz, C4, T4, T5, P3, Pz, P4, T6, O1, O2	Eyes-closed resting-state	With reference to individual alpha frequency (IAF): δ (IAF-8 to IAF-6 Hz), θ (IAF-6 to IAF-4 Hz), α1 (IAF-4 to IAF-2 Hz), α2 (IAF-2 to IAF Hz), α3 (IAF to IAF + 2 Hz), β1 (13–20 Hz), β2 (20–30 Hz)	Mean IAF peak lower in patients (M ± SEM = 9.4 ± 0.4 Hz) vs. controls (M ± SEM = 9.8 ± 0.3 Hz).With reference to IAF, decrease in amplitude of α2 and α3 (8–12 Hz) and general increase in δ sources amplitude in patients vs. controls. Lower α2 and α3 in patients in frontal, central, parietal, occipital, temporal, and limbic regions (α2 at Fz, C3, Cz, Pz and α3 at Fz, C3, Cz, C4, Pz). In controls, the maximal values of amplitude of α2 and α3 sources distributed in parieto-occipital regions. In patients, maximal values of amplitude of α2 and α3 sources distributed in posterior parietal and temporal areas Higher δ in central, parietal, occipital, and temporal regions (Fz, F4, C3, C4, O1, O2) in patients vs. controls No beta band differences reported	RD; selection bias; limited sample size
*Movement Paradigm Studies*												
Stępień et al. (2011)	CCr	Stroke patients (14) and age-matched controls (10)	Patients: M = 55.2 (range 41–68) Controls: M = 53 (range 43–68)	MRC upper limb strength: M = 4.4	Acute (2–13 days after onset)	Cortical (n = 5) and Subcortical (n = 9)	65 electrodes EEG	Fc3, FCz, Fc4, C5, C3, C1, Cz, C2, C4, C6, Cp3, CPz, Cp4	Externally paced mouse button press with L or R index finger	ERD strength in α1 (8–10 Hz), α2 (10–12 Hz), β1 (15–20 Hz), β2 (20–25 Hz) ERD percentage computed as (post–pre)/pre)*100, where post is the deepest amplitude decrease between -0.3 and 0.7 s relative to mouse button press and pre is a 0.5 s pre-stimulus interval	**Cortical group:** Interhemispheric α ERD asymmetries. Weaker DAM-H α ERD than CON-H α ERD when each H is contralateral to the acting hand. Stronger CON-H α ERD than DAM-H α ERD for paretic hand movement. Stronger contralateral α1 ERD for non-paretic (− 12.0%) than for paretic motor execution (− 7.4%). Decrease in α ERD amplitude in DAM-H pericentral sensorimotor areas (-9.5%) and CON-H (-14.3%) vs. controls (-29.2%) during contralateral hand movement **Subcortical group:** Symmetric α ERD between Hs. Stronger α ERD in DAM-H (-26.6%) than cortical stroke group (-9.5%). No significant differences in α ERD strength in DAM-H (-26.6%) or CON-H (-23.8%) vs. controls (-29.2%) for contralateral hand movement No differences found in the β frequency band for any of the main effects or interactions	RD; selection bias; small N of patients in each group; lesion descriptions not detailed
Tangwiriya-sakul et al. (2014)	CClr	Ischemic stroke patients (10) and age-matched controls (11)	Patients (M ± SD): 64.9 ± 13.4 Cortical group: M = 66 Subcortical group: M = 63.8 Controls: 57 ± 7.8	FM-UE at T0 cortical group: M = 36.6 FM-UE at T0 subcortical group: M = 41.6	Acute (within 7–14 days after onset) through chronic	Cortical (n = 5) and Subcortical (n = 5)	60 electrodes EEG	C3, C4, Cp3, Cp4	(1) Relax (idle) state during baseline movie (2) MA of movement seen in movement movie, lasting a minimum of 5 s* *Hand movie showed open/close L or R hand. A dominant hand movie shown to controls. A non-paretic hand movie shown to patients	ERD modulation strength *Sm* in μ (8–13 Hz) and β (15–25 Hz) at stroke onset (T0) and 1, 2, and 4 months post-stroke (T1, T2, T3, respectively) ERD calculated as percentage change between baseline and active (movement imagery or execution) *Sm* reflects both amplitude and spatial extent of ERD	DAM-H *Sm* absent or lower than CON-H *Sm* at T0 in most patients **Cortical group:** Absent or reduced baseline DAM-H μ and β components at T0 vs. controls. Absent or lower μ and β ERD modulation in DAM-H vs. CON-H at T0 for contralateral hand movement. Gradual re-appearance during post-stroke recovery. Larger H asymmetries vs. subcortical group. Difference between DAM-H (for paretic hand movement) and CON-H (for non-paretic hand movement) *Sm* larger in this group (-1.04) than in subcortical group (-0.25) or controls (0.13).* No-recovery cortical stroke patient showed no DAM-H *Sm* modulation, with CON-H *Sm* modulation increases during paretic hand movement **Subcortical group: **Slightly lower DAM-H μ and β ERD modulation vs. controls for contralateral hand movement *Negative values denote smaller DAM-H *Sm* than CON-H *Sm*	RD; selection bias; small N of patients in each group; lesion descriptions not detailed; patient attrition to follow-up (2 patients lost after T1 and 1 after T3)
Tang et al. (2020)	CClr	Ischemic stroke patients (22) and age-matched controls (25)	Patients: Mdn = 60 (range 55–62) Controls: Mdn = 59 (range 42–61)	Right-handed; FM-UE at enrolment: Mdn = 51 (range 43–60) ARAT at enrolment: Mdn = 36 (range 18–54)	Subacute (within 2 to 4 weeks after onset) through chronic	Subcortical: MCA territory, mostly CR and BG	Whole-head MEG	204 channels – planar gradiometer signals	Self-paced unilateral finger-lifting task	β (16–30 Hz) ERD and ERS amplitude and timing at 3, 5, and 12 weeks post-stroke (T1, T2, T3, respectively) ERD calculated as frequency-specific SMR power change during active period of movement relative to mean power of reference period (-3 to -2 s relative to movement onset) Peak ERD/ERS amplitudes determined in -2 to 2 s and 0 to 3 s, respectively	**Paretic hand movement at 3 weeks post-stroke:** Lower peak β ERD and ERS amplitudes in DAM-H M1 in patients vs. controls at T1. Earlier ERD activation in motor preparation phase (-700 ms, prior to onset at 0 ms) over PMc in CON-H in patients, compared to controls, which subsided during recovery. Prolonged bilateral β ERD and delayed β ERS at post-movement phase (900 ms), which shortened during recovery **Non-paretic hand movement:** Decreased β ERS peak amplitude in CON-H at 3 and 5 weeks, compared to controls, but not β ERD. No earlier ERD activation at 3 weeks post-stroke, but also delayed β ERS	RD; selection bias; possible handedness effect; patient attrition to follow-up (data available for 15 patients at T2 and for 16 at T3); ERD/ERS differences between patients and controls can be driven by changes in kinematics, but not likely
Gerloff et al. (2006)	CCr	Ischemic stroke patients (11) and controls (11)	Patients: Mdn = 70 (range 50–81) Controls: Mdn = 59 (range 48–79)	Right-handed; MRC paretic hand (M ± SD): 4.2 ± 0.4 (range 2–5) Substantial clinical recovery	Chronic (> 8 months after onset; Mdn = 2.5 years after onset; range 1–9)	Subcortical: left PLIC	28 electrodes EEG	Fc3, C3, Cp3, Fc4, C4, Cp4, Fz, FCz, Cz	(1) Rest: listening to metronome beats at 1 per second without moving (2) ME: metronome-paced extensions of fingers II–V at a rate of 1 per second with paretic hand	Task-related power in low α (8–10 Hz), high α (11–13 Hz), low β (16–20 Hz) and high β (22–26 Hz) Task-related spectral power calculated as a ratio between rest and activation	Reduced activation in DAM-H for paretic hand ME in high α (11–13 Hz), low β (16–20 Hz), and high β (22–26 Hz) in patients vs. controls Larger activation in task-related power in CON-H in patients vs. controls during paretic hand movement. Enhanced activity in low β (16–20 Hz) and high β (22–26 Hz) in CON-H extending from central region into frontal and prefrontal cortex in patients. In patients, the most pronounced enhancement of central region activity in CON-H found in low β (16–20 Hz) Differences at 8–10 Hz between patients and controls n.s	RD; selection bias; small sample size
Park et al. (2016)	CCr	Stroke patients (12*) and age-matched controls (12) *11 infarctions and 1 ICH	Patients (M ± SD): 54 ± 6.6 (range 45–70) SM1 + group: 51.3 ± 7.5 SM1- group: 56.8 ± 8.1 INF group: 54 ± 4.2 Controls: 57.8 ± 4.7	FMA-UE (M ± SD): 47.3 ± 9.2 SM1 + group: 47.8 ± 11.4 SM1- group: 54.3 ± 1.3 INF group: 40 ± 6.8	Chronic (M = 55.3 months after onset)	SM1 + : supratentorial cortico-subcortical, mostly MCA territory, including M1 (n = 4) SM1-: supratentorial subcortical, excluding M1; CR, thalamus, IC, BG (n = 4) INF: infratentorial, medial medulla or pons, without cerebellar damage) (n = 4)	64 electrodes EEG	28 electrodes around SMA and bilateral MC	Active, passive, and MI tasks with supination and grasping movements with paretic hand - only active and MI tasks analyzed* *In the passive task, a robotic device performs the movement. In the MI task, subjects are asked to imagine the movement with intention, without performing actual physical movement	Laterality coefficient (ERD/ERS hemispheric asymmetry index); topography in μ (8–13 Hz) and β (13–32 Hz) during a 2 s motor task period	**SM1 + group:** Higher CON-H β ERD/ERS in active and MI tasks than DAM-H **SM1- and INF groups:** Higher DAM-H β ERD/ERS in active and MI tasks than CON-H **SM1- group**: Stronger DAM-H β ERD than CON-H, but widespread during active supination movement **INF group:** Stronger DAM-H β ERD than CON-H focused on MC and parietal area during active supination movement. Stronger β ERD and more focused distribution in the DAM-H than that in the SM1- group. Similar beta band ERD/ERS power changes to controls Differences between patient sub-groups and controls n.s. in μ band	RD; selection bias; small N of patients in each group; lesion descriptions not detailed
Freudenburg et al. (2019)^††^	CCr	Ischemic stroke patient (1) and controls with severe refractory epilepsy (9)	Patient – 39; Age of controls not specified	Right-handed; LIS diagnosis, severely paralyzed	Chronic (13 years)	Pons (obliterated descending cortico-thalamic projections to rostral and caudal pontine nuclei that project to cerebellum via the MCP; but preserved PPN and DRTT)	Subdural ECoG	Bipolar electrode pairs over MC	(1) Baseline (rest): gazing at an image of a circle on a computer screen (2) Repetitive MA with right hand	Spectral content of baseline task. Block design: Spectral power decrease in LFB (6–30 Hz) during movement vs. rest; Spectral power increase in LFB during 3 s after movement (rebound) vs. rest	**Patient:** During rest, oscillatory α peak between 6–10 Hz, minimal modulation by attempted hand movement. On average, no distinct peaks in low frequencies above 10 Hz. Small LFB decrease (z-score close to 0) vs. controls (z-score = 2.14) and small LFB rebound (z-score = 1.04) in patient vs. controls during MA.* Some level of task-related modulation in the 6–10 Hz range. No task-related changes in the power of the β range (19–30 Hz) *The small LFB decrease and rebound during MA are accompanied by a task-induced clear HFB (> 30 Hz) response in 5 out of 6 electrode pairs. The centre frequency of the α peak may be lower than typically reported in EEG studies for alpha/mu band due to the usage of ECoG modality in this study	RD; selection bias; small sample size; subject-specific electrode locations
Höhne et al. (2014)^†††^	CSr	Ischemic stroke patient (1)	47	Tetraparetic, residual right-hand movement, can reliably write, type, or steer an electric wheelchair; residual eye movement and speech control; Daily usage of AT	Chronic	Pons	63 electrodes EEG	16 electrodes close to MC	MI of L hand and/or R hand and/or foot to execute a Copy task*. Then online feedback of targeted brain activation. If patient had control ≥ 70% in the Copy task phase: control of BCI application in Free mode *Copy task entails filling a matrix of free slots with coins of the same color in a game-based application	BCI system considers slow movement-related potentials, μ, β, δ ERD and β ERS Exact timing of features not specified	For patient MI, β ERS (rebound) and LRP are class-discriminant features for L vs. R hand. β ERS used to drive the system in 3 sessions. Consistent β ERS but differing spatial distribution across sessions. BCI accuracy increased within sessions, resulting in the most reliable control towards the end of each session. Imperfect control reached, but sufficient to drive the application in Free mode No consistent ERD component found in the α frequency band LRP feature prone to (eye) artifacts as patient featured involuntary eye movements, so not used	RD; selection bias; small sample size; discriminative power of each feature across sessions obtained with offline re-analysis of the Copy task data and global parameters (frequency band and time interval) chosen individually after manual inspection of data from all sessions; for each session, the same global parameters used, which may not be optimal
Kulasing-ham et al. (2022)	CClr	Ischemic stroke patients (9) and age-similar controls matched within five years (8)	Patients (M ± SD): 59.8 ± 15.7 Controls: 58 ± 13.1	Right-handed; mRS (M ± SD): 1 ± 0.5 (1^st^ visit) mRS (M ± SD): 0.5 ± 0.5 (2^nd^ visit) NIHSS (M ± SD): 0.7 ± 1.1 (1^st^ visit) NIHSS (M ± SD): 0.2 ± 0.4 (2^nd^ visit) Overall minor motor impairment	Acute to subacute (4–6 weeks after onset)	Cortico-subcortical: including BG, centrum semiovale, CR and some fronto-parietal, parieto-occipital and parietal cortex areas (n = 3) Subcortical: mostly thalamus, BG, CR, and PLIC (n = 5) Cortical: subcentral gyrus (n = 1)	Whole-head MEG	157 axial Gradiometers (Rolandic region analyzed)	(1) Baseline (rest): fixation on a cross projected onto a screen (2) Picture-word matching task with L or R button press	β (13–25 Hz) ERD and ERS ERD computed as the percentage decrease in average β power in time range of − 1 to 0.5 s relative to baseline (2 s relative to button press) ERS calculated as the percentage increase in average β power over the baseline in time range of 0.5 to 2.5 s	Lower relative β power for both picture-word matching and resting-state task in patients vs. controls at 1- and 6-months after stroke onset that did not improve over time. β power differences across Hs n.s Slightly lower β ERD in patients (M ± SD = 24.8 ± 9.6) vs. controls (M ± SD = 30 ± 9.4), but differences n.s. Lower β ERS in patients (M ± SD = 39.9 ± 27.7) vs. controls (M ± SD = 86.5 ± 44.3 Hz). Differences independent of lesion H ERD/ERS differences between DAM-H and CON-H n.s	RD; selection bias; small N of patients in each group

^**†**^Total study sample is larger (n = 18) with cortical lesions in all but one case. Only information regarding the stroke patients with cortical lesions is extracted

^**††**^Total study sample is larger, including another patient diagnosed with LIS due to ALS. Only information regarding the stroke patient (and/or vs. controls) is reported

^**†††**^Total study sample is larger, including another 2 patients with cerebral bleeding of unspecified origin and 1 patient with infantile cerebral palsy. Only information regarding the stroke patient is extracted

EEG electrode names and positions are reported in accordance with standard EEG nomenclature. Even numbers correspond to right hemisphere locations and odd numbers to left hemisphere locations. Lesion location descriptions exclude any asymptomatic lesions that have not affected patients’ clinical history and neurological examination, or that authors have not reported. In studies with several experimental conditions and/or outcomes of interest, only applicable information is reported. Statistical significance refers to *p* < .05

δ = delta band; θ = theta band; α = alpha band; μ = mu band; β = beta band; γ = gamma band; M = mean; Mdn = median; SD = standard deviation; SEM = standard error of the mean; T (time point post-stroke)

ALS (amyotrophic lateral sclerosis); ARAT (Action Research Arm Test; composed of 19 items categorized into grasp, grip, pinch and gross movement; range 0–4, where 0 denotes no movement and 3 denotes normally performed movement); AT (assistive technology); BG (basal ganglia); BMI (Brain-Machine-Interface); CClr (case–control restrospective design with longitudinal assessments); CCr (case–control retrospective design); CON-H (hemisphere contralateral to lesion); CR (corona radiata); CSr (cohort study with retrospective design); DAM-H (hemisphere ipsilateral to lesion); DAR (delta/alpha power ratio); DRTT (dentatorubrothalamic tract); DTABR (delta + theta)/(alpha + beta) ratio; ECoG (electrocorticography); EEG (electroencephalography); ERD (event-related desynchronization); ERS (event-related synchronization); FMA-UE (Fugl-Meyer Assessment of upper limb; range 0–66, where lower scores indicate more severe impairment, e.g., 66 indicates healthy function); H (hemisphere); HFB (high-frequency band); ICH (intracerebral hemorrhage); L (left); LFB (low-frequency band); LIS (locked-in syndrome); LRP (lateralized readiness potential); M1 (primary motor cortex); MA (movement attempt); MC (motor cortex); MCA (middle cerebral artery); MCP (middle cerebral peduncle); ME (motor execution); MI (motor imagery); MRC (Standard British Medical Research Council Motor Grading scale; range 2–5, with higher scores denoting better function); mRS (Modified Rankin Scale); N (number); NIHSS (National Institute of Health Stroke Scale; composed of 11 items each of which asseses a specific ability in a range 0–4, where a score of 0 reflects normal function in that ability and a higher score indicates some level of impairment); n.s. (not significant at *p* < .05); pdBSI (pairwise derived Brain Symmetry Index); PLIC (posterior limb of internal capsule); PM (premotor); PMc (premotor cortex); PoG (post-central gyrus); PPN (pedunculopontine nucleus); PrG (pre-central gyrus); PSD (power spectral density); R (right); RD (retrospective design); sec (seconds); SMA (supplementary motor area); SMR (sensorimotor rhythms); qEEG (quantitative electroencephalography); WM (white matter)

### Methodological quality of studies

Methodological quality assessment was performed at the included study level. Five publications fulfilled our criteria for high methodological quality, 7 were of moderate quality, and no studies were regarded as having low quality. Evaluation scores for each study can be consulted in Table 2.

**Table 2:** Quality scores of included studies (*n* = 12)

Author (year)	Study Participation	Study Attrition	Prognostic Factor Measurement	Outcome Measurement	Statistical Analysis and Reporting	Risk of Bias Score	Overall Quality Score
Van Wijngaarden et al. (2016)	Partial	Low	High	Low	Partial/ Low	Partial	Moderate quality
Finnigan et al (2016)	Partial	Low	Partial/ High	Low	Low	Partial	Moderate quality
Fanciullacci et al. (2017)	Partial	Low	Partial	Low	Low	Low	High quality
Babiloni et al. (2010)	Partial	Low	Partial	Low	Low	Low	High quality
Stępień et al. (2010)	High	Low	Partial/ High	Low	Partial	Partial	Moderate quality
Tangwiriyasakul et al. (2014)	Partial/ High	High	Partial	Partial	Partial	Partial	Moderatequality
Tang et al (2020)	Partial	Partial/ Low	Low	Low	Low	Low	High quality
Gerloff et al. (2006)	Partial	Low	Partial/ Low	Low	Low	Low	High quality
Park et al (2016)	Partial	Low	Partial	Low	Low	Low	High quality
Freudenburg et al. (2019)	High	Low	Low	Partial	Partial	Partial	Moderate quality
Höhne et al. (2014)	High	Partial/ Low	Partial	Low	Low	Partial	Moderate quality
Kulasingham et al. (2022)	High	Partial	Low	Low	Low	Partial	Moderate quality

Scores are based on the Quality in Prognosis Studies (QUIPS) tool. Low = Low risk of bias; Partial = Partial risk of bias; High = High risk of bias. Overall methodological quality: Low bias = High quality; Partial bias = Moderate quality; High bias = Low quality

A common methodological shortcoming across studies concerned the descriptions of the prognostic factor of interest – lesion location, which were overall of limited detail. This is why we could not rule out some bias in the ‘prognostic factor measurement’ quality domain. Another domain, which we considered as more heavily biased, was ‘study participation’. Reasons were the lack of reporting on key characteristics of the source population across articles, the inherent selection bias in identifying patients, and the mostly limited demographic information about the control groups. Furthermore, some investigations did not use the same method and setting of outcome measurement (e.g., the same electrophysiological monitoring technique and respective electrode selection) for all subjects, thus causing the respective ‘outcome measurement’ category to be downgraded. The response rate (‘study attrition’), analytical strategy, and presentation of results (‘statistical analysis and reporting’) were adequate in the majority of studies.

### Synthesis of evidence

We grouped studies according to the experimental paradigm they used, namely resting-state or movement-related, and decided to not inform our synthesis based on the assigned methodological quality scores. One reason was the small number of eligible articles and limited available data. Second, given the challenges in conducting research with stroke patients and the substantial heterogeneity found in these populations, we regarded the information extracted from studies with lower quality scores of significant theoretical value despite a higher risk of bias. A detailed narrative synthesis of each study’s principal findings is presented in Table 3.

**Table 3:** Narrative synthesis of included studies (*n* = 12)

Author (year)	Qualitative Description of Findings	
*Resting-state Studies*		
Van Wijngaarden et al. (2016)		Acute stroke patients with cortical lesions involving the MCA territory displayed the hallmarks of thalamo-cortical dysrhythmia (TCD): a characteristic downward shift of dominant alpha peaks in the resting-state EEG power spectra, together with increased power over the lower frequencies in the delta and theta range, when compared to control subjects. Contrary to general observations in TCD, the patients also exhibited a broad reduction in beta band activity. Using a biologically constrained model of a general thalamocortical module, authors showed that a lesion in the cortical component leads to sustained cell membrane hyperpolarization in the corresponding thalamic relay neurons. In turn, this switches these neurons from tonic spiking to a pathological bursting regime that results in the propagation of low-frequency oscillations beyond the restricted region of the lesion
Finnigan et al. (2016)		Acute stroke patients with cortical lesions comprising the MCA territory were compared to controls during eyes-closed resting-state in the relative bandpower of delta, theta, alpha, and beta, as well as in the following quantitative EEG indices that are sensitive to abnormal slow-wave (relative to faster) activity power: Delta/Alpha ratio, (delta + theta) / (alpha + beta) ratio, and Qslowing. The relative power of delta and theta was higher in patients, compared to controls, while the relative power of alpha and beta was lower in the patient group. All the respective EEG slowing indices were higher in patients than in controls, indicating a greater intensity of pathophysiological slow activity, relative to faster brain activity. Delta/Alpha ratio was found to be the index with the greatest capacity to discriminate between acute ischemic stroke and normative state, and 3.7 was identified as the optimal Delta/Alpha ratio threshold value for this purpose
Fanciullacci et al. (2017)		Subacute stroke patients with subcortical or cortico-subcortical lesions showed higher delta power across the whole scalp during eyes-closed resting-state, compared to controls, as measured using high density EEG. Cortico-subcortical stroke patients displayed lower alpha power compared to subcortical stroke patients across the entire scalp and in each hemisphere, as well as each studied area (bilateral frontal, central, and posterior areas). They also presented with a higher Delta/Alpha ratio compared to controls and compared to the subcortical group across the whole scalp and in the frontal and posterior areas on the contralesional (i.e., ipsilateral) side, where a higher Delta/Alpha value denotes a greater extent of EEG shift towards low-frequency activity. In subcortical stroke patients, lower delta power was found in the contralesional, compared to the ipsilesional (i.e., contralateral) hemisphere, as well as higher alpha power relative to the cortico-subcortical group
Babiloni et al. (2010)		Patients with LIS due to ventral pons injury demonstrated a decrease in the amplitude of alpha power in frontal, central, parietal, occipital, temporal, and limbic regions, and a general increase in the amplitude of delta sources during eyes-closed resting-state, compared to controls. The power of delta sources in central, parietal, occipital, and temporal areas was higher in patients with LIS, compared to controls, possibly due to an impaired neural synchronization mechanism between brainstem and cerebral cortex. No differences were discovered in the beta frequency band
*Movement Paradigm Studies*		
Stępień et al. (2011)		The ERD of alpha oscillations during externally paced index finger movements was lower in patients with acute cortical stroke over affected pericentral sensorimotor areas, compared to controls. Within the cortical stroke group, a smaller alpha ERD was documented in the ipsilesional hemisphere, compared to the contralesional hemisphere, when each was contralateral to the acting hand. When cortical stroke patients moved their paretic upper limb, the contralesional alpha ERD was stronger than the ipsilesional ERD. The alpha ERD amplitude on the lesioned side was relatively well-preserved for non-paretic hand movement, compared to the alpha ERD amplitude for paretic hand movement. In patients with subcortical strokes, symmetric alpha ERD was documented between hemispheres and the alpha ERD strength in the ipsilesional hemisphere was stronger than the one demonstrated in the cortical stroke group. No significant differences were shown between the ERD values in the subcortical group and controls during contralateral motor execution
Tangwiriyasakul et al. (2014)		Absence-or-reduction of ipsilesional mu and beta ERD modulation, compared to contralesional modulation, was reported in acute stroke patients with cortical and subcortical lesions at stroke onset during contralateral hand movement, in contrast to controls. Among the three groups (cortical, subcortical, and healthy), the largest difference between ipsilesional (during paretic movement) and contralesional (during non-paretic movement) activation was observed in the cortical stroke group, followed by the subcortical group, and nearly symmetric activation on both contralateral sides was revealed in control subjects during motor execution. In patients with subcortical strokes, the difference in the mean ERD modulation between the two hemispheres during paretic limb movement was not significant. Subcortical stroke patients also displayed an overall more bilateral ERD modulation during motor execution relative to the cortical stroke group. As patients were followed through to the chronic stage of stroke (at 1, 2, and 4 months), a trend of increasing ERD modulation on the ipsilesional side, accompanied by a decrease in the ERD modulation on the contralesional side, was found during recovery
Tang et al. (2020)		Patients studied in the subacute-through-chronic stage of stroke with subcortical lesions to the MCA territory, comprising mostly the corona radiata and basal ganglia, exhibited lower peak amplitudes of the movement-related beta ERD and ERS in the ipsilesional M1 during self-paced paretic finger movement at 3 weeks post-stroke, as well as reduced beta ERS in the contralesional hemisphere at 3- and 5-weeks post-stroke during non-paretic hand movement, compared to controls, as measured using whole-head MEG. Ipsilesional beta ERD correlated with functional motor recovery scores. In terms of the timing of the beta ERD/ERS during movement with the affected upper limb, there was earlier ERD activation in the motor preparation phase at -700 ms prior to movement onset over the pre-motor areas in the ipsilateral hemisphere, in comparison to controls, which subsided during recovery. A prolonged bilateral beta ERD was also shown, as well as delayed beta ERS at the post-movement phase around 900 ms after movement onset, which shortened during recovery. For non-paretic hand movement, no early beta ERD activation was documented at 3 weeks post-stroke in patients, but the beta ERS was delayed
Gerloff et al. (2006)		Well-recovered chronic stroke patients with subcortical lesions to the posterior limb of the left internal capsule showed reduced activation in the ipsilesional hemisphere during paretic hand finger movement in the alpha and beta frequency bands, compared to controls, as well as larger task-related beta activation in the contralesional hemisphere extending from the central region into the frontal and prefrontal cortex. Increased ipsilateral activity was suggested to facilitate control of recovered motor function by operating at a higher-order processing level, similar to the extended network concerned with complex movement in healthy individuals
Park et al. (2016)		Chronic stroke patients with supratentorial lesions that included M1 exhibited significantly greater beta band ERD/ERS intensity in the contralesional, compared to the ipsilesional hemisphere, during active and imagined paretic hand movement. In contrast, patients with supratentorial lesions that excluded M1, and infratentorial lesions to the medial medulla or pons, presented with the opposite pattern – higher beta ERD/ERS intensity on the ipsilesional, compared to the contralesional side. The group with supratentorial lesions, excluding M1, displayed higher beta ERD in the lesioned hemisphere during active supination movement, which was widespread, whereas the infratentorial lesion group demonstrated the increased ipsilesional beta ERD over the motor cortex and the parietal area. These findings were indicative of different hemispheric asymmetries and topographic characteristics of the beta band power according to the lesion. No significant differences between patient sub-groups and controls were discovered in the mu frequency band
Freudenburg et al. (2019)		A patient in the chronic phase of LIS caused by a brainstem stroke involving the pons, implanted with a fully implantable BCI, including subdural ECoG electrodes over the sensorimotor area, revealed atypical spectral power features associated with rest and movement. Small and inconsistent LFB (6–30 Hz) ERD and ERS were documented when the patient attempted to perform movement, in comparison with controls with refractory epilepsy who were implanted with subchronic implants with ECoG electrodes for diagnostic purposes. The oscillatory spectral power peaked between 6 and 10 Hz during rest, did not show any distinct peaks in low frequencies above 10 Hz, and the amplitude of the 6–10 Hz peak was hardly affected by attempted hand movement. No task-related changes were observed in the power range of the beta frequency band (19–30 Hz)
Höhne et al. (2014)		A tetraparetic patient who suffered a pontine stroke achieved control over a BCI system using beta ERS as a class-discriminant feature for left-hand versus right-hand motor imagery across six performed experimental sessions. Although the beta ERS was found consistently, the spatial distribution differed across sessions, requiring regular adaptation of the system to establish reliable external control. The BCI accuracy increased within sessions, exhibiting the most reliable control towards their respective termination. Another feature that was shown to be class-discriminant was the slow movement-related LRP, but in the end the LRP was not used due to its propensity to contamination from eye movements. No consistent ERD component was reported in the alpha frequency band
Kulasingham et al. (2022)		Minor stroke patients without significant hemiparesis or upper limb weakness with small cortico-subcortical, subcortical, and cortical lesions (n = 1) far from sensorimotor areas demonstrated lower beta band power during resting-state and lower beta band ERS during button press responses at 1- and 6-month post-stroke, compared to controls, which correlated with impaired bilateral motor dexterity and speed (i.e., slowed reaction times). Abnormalities persisted over visits and were observed in both hemispheres, consistent with the notion of bilateral motor impairments, independent of lesion location, suggestive of global network connectivity disruption affecting the sensorimotor cortex after minor stroke

BCI (Brain-Computer Interfaces); ECoG (electrocorticography); EEG (electroencephalography); ERD (event-related desynchronization); ERS (event-related synchronization); LFB (low-frequency band); LIS (locked-in syndrome); LRP (lateralized readiness potential); M1 (primary motor cortex); MCA (middle cerebral artery); MEG (magnetoencephalography); ms (millisecond); TCD (thalamo-cortical dysrhythmia)

Statistical significance refers to *p* < .05

#### Resting-state studies

Four investigations included resting-state paradigms (Table 1, section *Resting-state studies*) [31–34]. Four additional studies involved both a rest (baseline) and a movement-related task [35–38]. However, information concerning the rest task was discussed only in three of them [35, 37, 38].

#### Movement-related studies

Eight publications included movement-related conditions (Table 1, section *Movement-related studies*) [35–42]. Regarding the effort required for motor execution, as contingent upon subjects’ degree of motor impairment, and the performed motor tasks, studies employed the following: externally paced mouse button press in subjects with clear unilateral hemiparesis [39], open/close hand movement in patients with mostly moderate deficits [35], self-paced unilateral finger lifting in subjects with mild-to-moderate hand weakness [40], finger extension in patients with mild-to-moderate weakness, clumsiness, or spasticity in the affected hand [36], supination and grasping movements in individuals with unilateral motor problems that continued for a minimum of three months after their stroke [41], attempts to carry out repetitive hand movements in an individual with LIS [37], motor imagery of left or right hand used to play a computer-based game in a tetraparetic individual with some residual right-hand movement [42], and picture-word matching task with button press responses in minor stroke patients without significant hemiparesis or upper limb weakness [38]. Studies concentrated predominantly on the motor execution, rather than on the motor preparation time frame.

## Results

We performed a systematic review on the association between lesion location and SMR characteristics in stroke patients and grouped eligible studies according to the use of resting-state or movement-related experimental paradigms.

### SMR Characteristics in resting-state studies

In studies that assessed SMR characteristics using resting-state paradigms, a predominant pattern was found of an overall decrease in alpha (∼8–12 Hz) and beta (~ 13–30 Hz) and increase in delta (~ 1–4 Hz) spectral power in patients with cortical or pontine injury, compared to controls, whereas findings for subcortical stroke were mixed. To illustrate, Van Wijngaarden et al. (2016) documented lower alpha and higher delta band power in acute stroke patients with cortical lesions encompassing the middle cerebral artery (MCA) territory, relative to healthy volunteers [31]. Similarly, Finnigan et al. (2016) assessed several EEG indices indicative of pathophysiological slow-wave activity, such as the delta/alpha and (delta + theta)/(alpha + beta) power ratio and discovered that they were higher across the scalp in cortical stroke patients than in controls [32]. In addition, Tangwiriyasakul and colleagues (2014) discovered absent or reduced 8–13 Hz and 15–25 Hz components in the baseline EEG in subjects with cortical damage in the ipsilesional hemisphere, which was not observed in controls [35].

Two studies reported on patients with subcortical or mixed (subcortical with cortical involvement) stroke. Fanciullacci et al. (2017) demonstrated a shift towards low-frequency activity in subacute stroke patients with mixed or subcortical lesions, compared to controls. The mixed group exhibited higher delta/alpha ratio compared to the subcortical group and lower alpha power over each hemisphere and the whole scalp [33]. In contrast, a recent study on a group of stroke patients with small lesions far from sensorimotor areas (predominantly subcortical, some mixed, and 1 cortical), and bilateral, but minor, impairments in motor dexterity and speed showed reduced baseline beta power in patients, relative to healthy volunteers, that was independent of lesion hemisphere or location [38].

Furthermore, two investigations included chronic stroke patients with LIS caused by pontine injury. Babiloni et al. (2010) showed a decrease in alpha and a general increase in delta power over central, parietal, occipital, and temporal regions in patients with ventral pons lesions, relative to controls [34]. The other study by Freudenburg and colleagues (2019) examined a single subject whose lesion obliterated the descending cortico-thalamic projections to the rostral and caudal pontine nuclei. The patient displayed an alpha oscillatory peak between 6–10 Hz, as measured with subdural ECoG electrodes over the sensorimotor area, the amplitude of which was not compared to that of controls [37].

The authors referred to several mechanisms to explain their findings. Resting-state alpha rhythms of regular amplitude denote a healthy brain prepared to process information [43], whereas thalamic-generated delta rhythms characterize slow-wave sleep [44]. The balance between these rhythms is determined by glutamatergic, cholinergic, and/or GABAergic inputs from cortical, thalamic, and brainstem modules. Disruptions to thalamo-cortical circuits, due to for instance cortical [31] or pontine [37] lesions could interfere with the interactions of these systems and drive thalamic nuclei into a mode of pathological low-frequency bursting [45]. The stroke-induced pathological oscillations may propagate beyond the primary lesion and alter the physiological state of remote, but functionally connected brain regions, consistent with the notion of *diaschisis* [46].

### SMR characteristics in movement-related studies

To interpret the findings from movement-related studies in stroke patients, it is important to outline the ERD patterns related to healthy voluntary movement in people without motor impairment or stroke. It has been shown that a circumscribed alpha and beta desynchronization occurs approximately 2 s prior to movement onset over the contralateral Rolandic region. The desynchronization typically becomes bilaterally symmetrical promptly before, as well as during the actual movement execution [47–49]. Movement-related alpha and beta ERD generated from precentral areas, as well as areas posterior to the central sulcus, and in both contralateral and ipsilateral sensorimotor cortices, have also been revealed using ECoG measured from able-bodied individuals with epilepsy [17].

The studies reviewed here presented converging evidence for alpha and beta ERD attenuation over the ipsilesional hemisphere and for interference of stroke with the balance between ipsilateral and contralateral ERD. Although this appears to be a recurrent pattern, cortical and subcortical damage seem associated with different levels of effect on the SMRs.

#### Cortical stroke

In cases of lesions with cortical involvement, alpha and beta ERD amplitudes over the ipsilesional hemisphere associated with paretic hand movement, were substantially reduced, compared to those in controls. To illustrate, Stępień et al. (2011) found decreased alpha ERD amplitude in an acute cortical stroke group in the ipsilesional hemisphere during contralateral hand movement, relative to healthy subjects [39]. Comparable results were reported by Tangwiriyasakul et al. (2014) in the mu and beta bands, who examined patients at 1, 2, and 4 months after stroke onset and found absent or reduced mu and beta ERD on the ipsilesional side shortly after stroke onset and a recovery of this feature in the course of time [35]. In no-recovery individuals with cortical injury [35], including in the chronic stage [41], ipsilesional ERD appears to remain dampened.

In the contralesional hemisphere, larger ERD was observed than in the ipsilesional hemisphere, both in the acute [35, 39] and chronic [41] phase of stroke. During recovery, a decreasing trend in contralesional ERD modulation was revealed, whereas the opposite tendency was documented in no-recovery cases [35].

#### Subcortical stroke

The influence of subcortical lesions on ipsilesional ERD appeared variable. In the (sub)-acute stage of stroke, this effect ranged from no substantial differences in ERD strength in comparison with controls during contralateral hand movement [35, 38, 39] to substantially diminished alpha and beta ERD amplitudes [40], Despite these inconsistencies, the two investigations that directly compared patients with cortical and subcortical injury were similar in reporting less pronounced effects of subcortical than of cortical lesions on ERD modulation [35, 39]. These observations agreed with those of Park and colleagues for chronic stroke [41]. In their study, individuals with stroke encompassing subcortical and cortical (including M1) regions demonstrated lower beta ERD/ERS on the ipsilesional, compared to contralesional side, during active and imagined movement. In comparison, in subcortical strokes that excluded M1, and infratentorial strokes, the same authors reported an opposite trend, with more widespread contralateral activation, which resembled more closely the tendency in controls. Others reported ipsilesional ERD increases in the course of recovery [35, 40], although persistently attenuated activation in the lesioned hemisphere into the chronic phase of stroke was also observed [36, 41].

On the side opposite to the lesion, ERD was not found to be significantly affected in the acute stage post-stroke [39, 40]. Interestingly, two studies examined moderately [40] or mildly, but bilaterally impaired stroke patients with mostly subcortical lesions [38] and documented a significant decrease in beta ERS in both the lesioned and the non-lesioned hemisphere [38, 40], indicating bilaterally altered Rolandic beta band activity. In later stages after injury, Tangwiriyasakul et al. discovered a pattern of decreasing contralesional ERD with reappearance of ipsilesional modulation in patients with better recovery [35], whereas Gerloff et al. noted larger ipsilateral activation in task-related power during movement of the affected limb in patients compared to controls [36].

#### Pontine stroke

Concerning pontine stroke, mixed evidence was revealed. To illustrate, Freudenburg et al. (2019) demonstrated minimal alpha modulation related to attempted hand movement in an individual with LIS due to brainstem stroke and excluded impaired task performance as a likely reason. Beta power changes linked to motor execution were practically absent [37]. On the other hand, Höhne and colleagues (2014) tested the applicability of an EEG-based BCI system for control that could be driven by a wide range of spectral features, such as mu, beta, and delta desynchronization, or beta synchronization. While no consistent ERD component was found in the alpha band, beta ERS was determined as a reliable EEG feature, which enabled a tetraparetic individual to gain control over the BCI. Of note, the beta synchronization displayed differing spatial distribution across sessions and regular calibration of the system was needed to maintain reliable decoding [42]. Interestingly, in contrast to the results from these two investigations, the infratentorial lesion group assessed by Park et al. (2016) whose lesions comprised the medial medulla or pons, exhibited similar beta band ERD and ERS power changes to controls with a stronger ipsilesional than contralesional beta desynchronization [41]. The apparent discrepancy could be related to the degree of motor impairment (moderate for the participants of Park et al. [41] versus severe for those of Freudenburg [37] and Höhne [42]). Nevertheless, data from these examinations are based on six patients and any firm conclusions remain to be confirmed in larger samples.

**Fig. 2:**
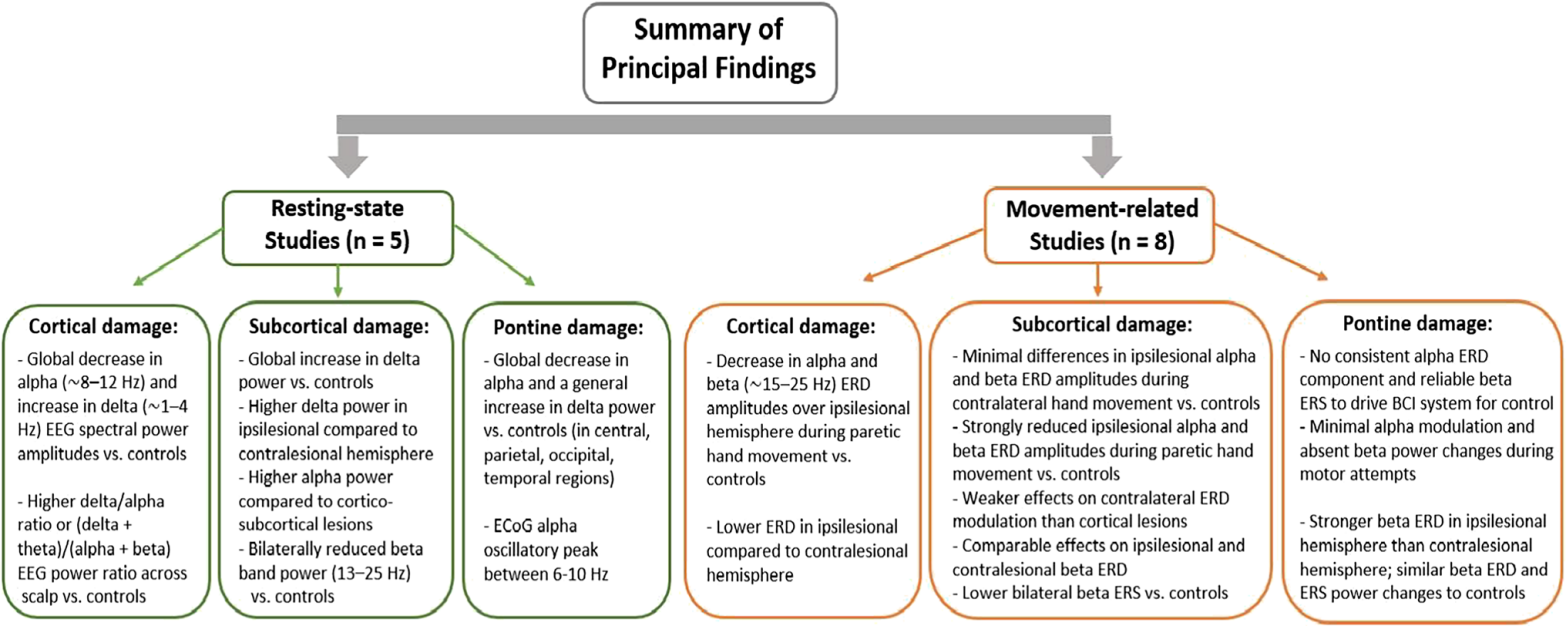
Main study findings as per data synthesis according to experimental paradigm. *Note.* N refers to the number of studies that findings are based on in each category. ECoG (electrocorticography); EEG (electroencephalography); ERD (event-related desynchronization); ERS (event-related synchronization)

In summary, we established a common trend of global resting-state decreases in alpha power in individuals with cortical or pontine injury. Notably, the number of studies investigating resting-state phenomena was low, subcortical stroke in particular was rarely addressed, and the lesion location descriptions varied in detail. Therefore, further research is needed to gain full understanding of the resting-state SMRs. In movement-related studies, both cortical and subcortical strokes resulted in alpha and beta ERD attenuation over the contralateral hemisphere during paretic hand movement in patients relative to controls. Cortical damage was associated with significant ipsilesional decreases in alpha and beta ERD. Subcortical injury, on the other hand, appeared to interfere with ERD to a lesser extent than cortical stroke in direct comparisons between these populations, but results across studies on subcortical populations alone were highly variable. Regarding pontine strokes, results were inconclusive and based on a small number of cases, so more research is necessary to support our interpretations. Figure 2 provides a visual summary of the main tendencies demonstrated across studies.

## Discussion

An important contribution of this study is that, to the best of the authors’ knowledge, it represents the first systematic examination of the association between lesion location and SMR characteristics in stroke patients. Other strong aspects involve the exhaustive methodological approach with a reproducible search strategy, as well as the pre-registration and adherence to a published protocol and the PRISMA guidelines.

A limitation of this review is the substantial variability across publications. This prevented us from performing a formal meta-analysis, which would have enabled a more precise delineation of the relationship between lesion location and SMRs. Indeed, lesion location reporting was inconsistent, and descriptions were insufficiently detailed with most authors categorizing lesions only as either cortical or subcortical. In addition, some studies assessed a single group of patients with the same stroke damage, while others reported on multiple sub-groups of patients with different lesion locations. While common signal alterations were demonstrated in patients relative to controls, the analysis of subsets of patients according to lesion location revealed important differences in the resulting SMRs. Another source of variation in this review was the focus on either interhemispheric differences or comparisons between groups, as well as the sole examination of paretic hand movement in some investigations and contralateral patterns of both hands in others. Furthermore, there was large heterogeneity in patients’ motor deficit at stroke onset, which also relates to the type of movement and level of effort required to comply with the performed motor tasks in the movement paradigm studies. This leaves a possibility that some of the presented findings could be driven by subjects with poorer motor skill or recovery. Patients with very limited-to-no residual ability in their affected limb may rely on different mechanisms during overt motor performance, compared to healthy volunteers or less severely affected individuals, such as for instance engaging in motor imagery alongside the execution component [50], which conceptually could result in different cortical activation patterns. Thus, a consideration of lesion topography, as well as level of motor impairment, may provide a more exhaustive account of our results. In this regard, the findings of Kulasingham et al. [38] of decreased beta ERD and ERS in unilateral minor stroke patients with mild bilateral motor impairment, independent of lesion location, are worth mentioning. The authors argued that the observed bilateral deficits could reflect a more global stroke-induced disruption, not tied to lesion location, involving bilateral excitation/inhibition imbalance, bilateral modulation of motor planning and execution [51, 52], and disturbance of global network connectivity. Interestingly, bilateral cortical abnormalities, independent of lesion location, have previously been documented using functional magnetic resonance imaging (fMRI) following subcortical stroke [53–55]. In addition, the dynamic patterns of the cortical changes have been linked to functional connectivity between cortical motor areas remote from the infarct [54, 55] and parieto-frontal networks integrating visuomotor information [56], as well as impairment of specific subsets of the corticospinal tract (CST) and patients’ corresponding degree of recovery [53]. Such results suggest the possibility that lesion location is a determinant of SMR changes in the context of greater motor disability, including hemiparetic symptomatology, especially if the motor cortex is damaged, whereas a more globally altered connectivity could be the mechanism underlying minor strokes that lack severe motor disability and cortical features. These findings encourage the further exploration of the relationship between stroke lesion location and milder forms of motor impairment in future studies, with motor spasticity being a particularly interesting symptom, given its relevance to long-term clinical recovery [57], as well as contrasting reports regarding its relationship to ipsilesional ERD [58]. Another promising direction for future research is to complement electrophysiological measurements with lesion network mapping and functional connectivity analyses in order to identify the broader connectivity patterns of distinct lesion locations and their association with different clinical phenotypes [57]. This can provide greater insight into the impact of different lesion locations on both local and global properties of the intrinsic motor system [56, 57] and unravel the mechanisms by which even remotely localized lesions can directly impair the integrity of the input and output fibers connected with the sensorimotor cortex [53] and potentially impact subjects’ ability for volitional SMR modulation [56]. Lastly, the majority of studies suffered from inadvertent selection bias, did not offer exhaustive descriptions of their samples, particularly of the controls, and did not systematically identify potential confounders or formally adjust for them in their analyses. Including larger and more homogeneous patient populations, more detailed demographic information, as well as details on potential study confounders, and reporting anatomical lesion location more precisely in future examinations would help determine the robustness of the presented results. It would also be beneficial to standardize frequency band definitions and outcome measures in the field to enable more comprehensible cross-study comparison and synthesis of future research findings, and to facilitate research translation into clinical practice.

To summarize, this systematic examination partially supports the notion that SMR patterns in stroke patients differ according to lesion location in that cortical involvement in a lesion seems associated with more severe SMR alterations. In addition, recovery of ERD over time following stroke appears to relate to improvements in motor function. In line with this premise, literature has suggested a link between the strength and laterality of the ipsilesional desynchronization in the initial months after injury and better motor outcomes [21]. Therefore, ERD modulation at the acute and subacute post-stroke stages may be investigated as a potential motor recovery biomarker or as a tool to stratify patients who may benefit more from (certain) rehabilitative therapies. Furthermore, the revealed ipsilesional ERD reductions, particularly in patients with cortical damage, could inform the design of BCIs that can promote motor recovery by guiding patients to activate their lesioned hemisphere and prevent learned non-use [59]. Future investigations should clarify the extent to which BCI interventions can support recovery of ipsilesional ERD modulation and if so, what causal effects could be expected on functional outcomes. Our findings also set a foundation for developing more effective and personalized BCIs. For instance, BCIs may use neuroelectrical signals extracted from the contralesional hemisphere when decoding movement intention of patients with cortical damage to improve decoding performance. Another future step in developing patient-tailored treatment approaches and BCI solutions is a further exploration of how factors, such as differences in lesion size, severity, and distance to sensorimotor areas [38], brain structure [60] and functional network integrity [38, 56], or cognitive strategies [61], may influence subjects’ ability to modulate their brain activity.

## Conclusion

We systematically reviewed the literature on the association between lesion location and SMRs in stroke patients. Despite the heterogeneity across studies and their methodological limitations, the data suggest that different lesion locations are associated with distinct levels of alterations in resting-state and movement-related sensorimotor signals. Based on our investigation, we argue that damage to cortical structures, particularly the motor cortex, relates to the severity of SMR changes following stroke, whereas subcortical damage may impact SMR modulation due to alterations in functional network integrity. We underscore the need of future examinations to address the shortcomings of current research to enable further elucidation of the role of lesion location on SMRs. We envision that this can contribute to patient stratification for neurorehabilitation protocols and improve our understanding of patients’ likelihood to benefit from assistive and rehabilitative BCIs.

### Supplementary information

Below is the link to the electronic supplementary material.

Supplementary file1 (DOCX 32 KB)

## Data Availability

Not applicable.

## Abbreviations

BCI: Brain-Computer Interface
ECoG: Electrocorticography
EEG: Electroencephalography
EMBASE: Excerpta Medica Database
ERD: Event-related desynchronization
ERS: Event-related synchronization
HFB: High-frequency band
LFB: Low-frequency band
M1: Primary motor cortex
MEG: Magnetoencephalography
PRISMA: Preferred Reporting Items for Systematic Reviews and Meta-Analyses
PROSPERO: International Prospective Register of Systematic Reviews
QUIPS: Quality in Prognosis Studies
SMRs: Sensorimotor rhythms
