# Supplementary material for: Association between lesion location and sensorimotor rhythms in stroke – a systematic review with narrative synthesis
Source: Neurol Sci. 2023 Aug 22;44(12):4263–89. doi: 10.1007/s10072-023-06982-8 (PMC10641054; doi:10.1007/s10072-023-06982-8)
Supplement: Supplementary file 1 — Supplementary file1 (DOCX 32 KB) [file 10072_2023_6982_MOESM1_ESM.docx]

**Neurological Sciences**

**Association Between Lesion Location and Sensorimotor Rhythms in Stroke - A Systematic Review with Narrative Synthesis**

Ivana Kancheva, MA, Sandra M.A. van der Salm, MD, PhD, Nick F. Ramsey, PhD,

Mariska J. Vansteensel, PhD

*UMC Utrecht Brain Center, Department of Neurology & Neurosurgery, University Medical Center Utrecht, Utrecht, The Netherlands*

**Corresponding author:** Dr. Mariska J Vansteensel, UMC Utrecht Brain Center, Department of Neurology & Neurosurgery, University Medical Center Utrecht, P.O. Box 85060, 3508 AB Utrecht, The Netherlands, Phone +31 88 7555121, [m.j.vansteensel@umcutrecht.nl](mailto:m.j.vansteensel@umcutrecht.nl).

**Supplementary Information**

| **Supplementary Table 1.** Full electronic search strategy per database |
| --- |
| **Full PubMed Strategy**  ((((stroke*[Title/Abstract] OR infarct*[Title/Abstract] OR cortical[Title/Abstract] OR subcortical[Title/Abstract] OR infratentorial[Title/Abstract] OR supratentorial[Title/Abstract] OR acute[Title/Abstract] OR subacute[Title/Abstract] OR chronic[Title/Abstract] OR mesencephal*[Title/Abstract] OR midbrain[Title/Abstract] OR brainstem[Title/Abstract] OR pontine[Title/Abstract] OR pons[Title/Abstract] OR cerebral[Title/Abstract] OR brain[Mesh] OR lesion*[Title/Abstract]) AND (locat*[Title/Abstract] OR site*[Title/Abstract])) AND (brain wave*[Title/Abstract] OR brainwave*[Title/Abstract] OR alpha[Title/Abstract] OR beta[Title/Abstract] OR mu[Title/Abstract] OR delta[Title/Abstract] OR "Brain Waves"[Mesh:NoExp] OR "Alpha Rhythm"[Mesh] OR "Beta Rhythm"[Mesh] OR "Delta Rhythm"[Mesh] OR sensorimotor[Title/Abstract] OR sensory[Title/Abstract] OR somatosensory[Title/Abstract] OR motor[Title/Abstract] OR motor cort*[Title/Abstract] OR cort*[Title/Abstract] OR movement-related[Title/Abstract] OR pre-movement related[Title/Abstract] OR movement intention[Title/Abstract] OR movement execution[Title/Abstract] OR motor imagery[Title/Abstract] OR "Sensorimotor Cortex"[Mesh:NoExp] OR "Motor Cortex"[Mesh] OR "Somatosensory Cortex"[Mesh] OR electrophysiol*[Title/Abstract] OR electrical[Title/Abstract] OR low-frequency[Title/Abstract] OR brain[Title/Abstract] OR neural[Title/Abstract] OR baseline[Title/Abstract] OR spectral[Title/Abstract] OR power spectral[Title/Abstract] OR rest*[Title/Abstract] OR eyes-closed[Title/Abstract])) AND (event-related desynchron*[Title/Abstract] OR ERD[Title/Abstract] OR ERS[Title/Abstract] OR event-related synchron*[Title/Abstract] OR cortical synchron*[Title/Abstract] OR cortical phase synchron*[Title/Abstract] OR cortical desynchron*[Title/Abstract] OR cortical phase desynchron*[Title/Abstract] OR rebound*[Title/Abstract] OR "Cortical Synchronization"[Mesh] OR potential*[Title/Abstract] OR rhythm*[Title/Abstract] OR correlate*[Title/Abstract] OR substrate*[Title/Abstract] OR signal*[Title/Abstract] OR biomarker*[Title/Abstract] OR pattern*[Title/Abstract] OR response*[Title/Abstract] OR amplitude*[Title/Abstract] OR recording*[Title/Abstract] OR feature*[Title/Abstract] OR densit*[Title/Abstract] OR recover*[Title/Abstract] OR oscillat*[Title/Abstract] OR activation*[Title/Abstract] OR activity[Title/Abstract] OR excitability[Title/Abstract] OR alteration*[Title/Abstract] OR modulation*[Title/Abstract])) AND (stroke*[Title/Abstract] OR "Stroke"[Mesh] OR LIS[Title/Abstract] OR locked-in syndrome[Title/Abstract] OR paralys*[Title/Abstract] OR hemiparalys*[Title/Abstract] OR hemipare*[Title/Abstract] OR paretic[Title/Abstract] OR paresis[Title/Abstract] OR parapleg*[Title/Abstract] OR hemipleg*[Title/Abstract] OR quadripleg*[Title/Abstract] OR motor impair*[Title/Abstract] OR spastic*[Title/Abstract] OR "Paresis"[Mesh] OR "Paralysis"[Mesh:NoExp] OR "Locked-In Syndrome"[Mesh] OR "Hemiplegia"[Mesh] OR "Paraplegia"[Mesh] OR ''quadriplegia''[Mesh]) |
| **Full Embase Strategy**  (stroke*:ti,ab,kw OR infarct*:ti,ab,kw OR cortical:ti,ab,kw OR subcortical:ti,ab,kw OR infratentorial:ti,ab,kw OR supratentorial:ti,ab,kw OR acute:ti,ab,kw OR subacute:ti,ab,kw OR chronic:ti,ab,kw OR mesencephal*:ti,ab,kw OR midbrain:ti,ab,kw OR pontine:ti,ab,kw OR pons:ti,ab,kw OR lesion*:ti,ab,kw OR brain:ti,ab,kw OR 'brain lesion*':ti,ab,kw OR 'cerebral lesion*':ti,ab,kw OR 'brain'/exp OR 'brain damage'/exp OR 'cerebrovascular accident'/exp) AND (locat*:ti,ab,kw OR site*:ti,ab,kw) AND ('brain wave*':ti,ab,kw OR brainwave*:ti,ab,kw OR 'alpha rhythm*':ti,ab,kw OR 'beta rhythm*':ti,ab,kw OR 'delta rhythm*':ti,ab,kw OR 'mu rhythm*':ti,ab,kw OR 'electroencephalogram'/exp OR sensorimotor:ti,ab,kw OR sensory:ti,ab,kw OR somatosensory:ti,ab,kw OR motor:ti,ab,kw OR 'motor cort*':ti,ab,kw OR cort*:ti,ab,kw OR 'movement related':ti,ab,kw OR 'pre-movement related':ti,ab,kw OR 'movement intention':ti,ab,kw OR 'movement execution':ti,ab,kw OR 'motor imagery':ti,ab,kw OR 'sensorimotor cortex':ti,ab,kw OR 'motor cortex':ti,ab,kw OR 'somatosensory cortex':ti,ab,kw OR 'sensorimotor cortex'/exp OR 'somatosensory cortex'/exp OR 'motor cortex'/exp OR electrophysiol*:ti,ab,kw OR electric*:ti,ab,kw OR 'low frequency':ti,ab,kw OR brain:ti,ab,kw OR neural:ti,ab,kw OR baseline:ti,ab,kw OR evoked:ti,ab,kw OR spectral:ti,ab,kw OR 'power spectral':ti,ab,kw OR rest*:ti,ab,kw OR 'eyes closed':ti,ab,kw) AND ('event-related desynchron*':ti,ab,kw OR ERD:ti,ab,kw OR ERS:ti,ab,kw OR 'event-related synchron*':ti,ab,kw OR 'cortical synchron*':ti,ab,kw OR 'cortical phase synchron*':ti,ab,kw OR 'cortical desynchron*':ti,ab,kw OR 'cortical phase desynchron*':ti,ab,kw OR 'cortical synchronization'/exp OR 'cortical excitability'/exp OR 'evoked response'/exp OR rebound*:ti,ab,kw OR potential*:ti,ab,kw OR rhythm*:ti,ab,kw OR correlate*:ti,ab,kw OR substrate*:ti,ab,kw OR signal*:ti,ab,kw OR biomarker*:ti,ab,kw OR pattern*:ti,ab,kw OR response*:ti,ab,kw OR amplitude*:ti,ab,kw OR recording*:ti,ab,kw OR feature*:ti,ab,kw OR densit*:ti,ab,kw OR recover*:ti,ab,kw OR oscillat*:ti,ab,kw OR activation*:ti,ab,kw OR activity:ti,ab,kw OR excitability:ti,ab,kw OR alteration*:ti,ab,kw OR modulation*:ti,ab,kw) AND (stroke*:ti,ab,kw OR 'stroke sufferer*':ti,ab,kw OR 'stroke patient'/exp OR LIS:ti,ab,kw OR 'locked-in syndrome':ti,ab,kw OR 'locked in syndrome'/exp OR paralys*:ti,ab,kw OR hemiparalys*:ti,ab,kw OR 'paralysis'/mj OR hemipare*:ti,ab,kw OR paretic:ti,ab,kw OR paresis:ti,ab,kw OR 'hemiparesis'/exp OR 'paresis'/exp OR parapleg*:ti,ab,kw OR hemipleg*:ti,ab,kw OR 'paraplegia'/exp OR 'hemiplegia'/exp OR quadripleg*:ti,ab,kw OR 'quadriplegia'/exp OR 'motor dysfunction*':ti,ab,kw OR 'motor impair*':ti,ab,kw OR spastic*:ti,ab,kw)  *Note.* Search results were extracted on May 6^th^, 2021. No published search filters were used. |

**Supplementary Table 2.** Preferred Reporting Items for Systematic Reviews and Meta-Analyses (PRISMA) statement

| **Section and Topic** | **Item #** | **Checklist item** | **Reported Yes/No (location where item is reported)** |
| --- | --- | --- | --- |
| **TITLE** | | |  |
| Title | 1 | Identify the report as a systematic review. | Yes |
| **ABSTRACT** | | |  |
| Abstract | 2 | See the PRISMA 2020 for Abstracts checklist. | Yes |
| **INTRODUCTION** | | |  |
| Rationale | 3 | Describe the rationale for the review in the context of existing knowledge. | Yes, section ‘Introduction’ |
| Objectives | 4 | Provide an explicit statement of the objective(s) or question(s) the review addresses. | Yes, section ‘Introduction’ |
| **METHODS** | | |  |
| Eligibility criteria | 5 | Specify the inclusion and exclusion criteria for the review and how studies were grouped for the syntheses. | Yes, section ‘Methods’, sub-section ‘Search Strategy and Selection Criteria’ |
| Information sources | 6 | Specify all databases, registers, websites, organisations, reference lists and other sources searched or consulted to identify studies. Specify the date when each source was last searched or consulted. | Yes, section ‘Methods’, sub-section ‘Search Strategy and Selection Criteria’; full electronic search strategy per database reported in Supplementary Figure 1 |
| Search strategy | 7 | Present the full search strategies for all databases, registers, and websites, including any filters and limits used. | Yes, full electronic search strategy per database reported in Supplementary Figure 1 |
| Selection process | 8 | Specify the methods used to decide whether a study met the inclusion criteria of the review, including how many reviewers screened each record and each report retrieved, whether they worked independently, and if applicable, details of automation tools used in the process. | Yes, section ‘Methods’, sub-section ‘Search Strategy and Selection Criteria’ |
| Data collection process | 9 | Specify the methods used to collect data from reports, including how many reviewers collected data from each report, whether they worked independently, any processes for obtaining or confirming data from study investigators, and if applicable, details of automation tools used in the process. | Yes, section ‘Methods’, sub-section ‘Search Strategy and Selection Criteria’, main-text Figure 1 |
| Data items | 10a | List and define all outcomes for which data were sought. Specify whether all results that were compatible with each outcome domain in each study were sought (e.g., for all measures, time points, analyses), and if not, the methods used to decide which results to collect. | Yes, section ‘Methods’, sub-section ‘Data Extraction’; Table 1 |
|  | 10b | List and define all other variables for which data were sought (e.g., participant and intervention characteristics, funding sources). Describe any assumptions made about any missing or unclear information. | Yes, section ‘Methods’, sub-section ‘Data Extraction’; Table 1 |
| Study risk of bias assessment | 11 | Specify the methods used to assess risk of bias in the included studies, including details of the tool(s) used, how many reviewers assessed each study and whether they worked independently, and if applicable, details of automation tools used in the process. | Yes, section ‘Methods’, sub-section ‘Methodological Quality Assessment’ |
| Effect measures | 12 | Specify for each outcome the effect measure(s) (e.g., risk ratio, mean difference) used in the synthesis or presentation of results. | n/a |
| Synthesis methods | 13a | Describe the processes used to decide which studies were eligible for each synthesis (e.g., tabulating the study intervention characteristics and comparing against the planned groups for each synthesis (item #5)). | Yes, section ‘Methods’, sub-sections ‘Data Synthesis’ and ‘Synthesis of Evidence’ |
|  | 13b | Describe any methods required to prepare the data for presentation or synthesis, such as handling of missing summary statistics, or data conversions. | n/a |
|  | 13c | Describe any methods used to tabulate or visually display results of individual studies and syntheses. | Yes, section ‘Methods’, sub-section ‘Data Synthesis’ |
|  | 13d | Describe any methods used to synthesize results and provide a rationale for the choice(s). If meta-analysis was performed, describe the model(s), method(s) to identify the presence and extent of statistical heterogeneity, and software package(s) used. | Yes, section ‘Methods’, sub-section ‘Data Synthesis’ |
|  | 13e | Describe any methods used to explore possible causes of heterogeneity among study results (e.g., subgroup analysis, meta-regression). | n/a |
|  | 13f | Describe any sensitivity analyses conducted to assess robustness of the synthesized results. | n/a |
| Reporting bias assessment | 14 | Describe any methods used to assess risk of bias due to missing results in a synthesis (arising from reporting biases). | n/a |
| Certainty assessment | 15 | Describe any methods used to assess certainty (or confidence) in the body of evidence for an outcome. | n/a |
| **RESULTS** | | |  |
| Study selection | 16a | Describe the results of the search and selection process, from the number of records identified in the search to the number of studies included in the review, ideally using a flow diagram. | Yes, section ‘Methods’, sub-section ‘Selection of Studies’; main-text Figure 1 |
|  | 16b | Cite studies that might appear to meet the inclusion criteria, but which were excluded, and explain why they were excluded. | n/a |
| Study characteristics | 17 | Cite each included study and present its characteristics. | Yes, Table 1 |
| Risk of bias in studies | 18 | Present assessments of risk of bias for each included study. | Risk of bias/methodological quality assessment of studies presented in Table 2 |
| Results of individual studies | 19 | For all outcomes, present, for each study: (a) summary statistics for each group (where appropriate) and (b) an effect estimate and its precision (e.g., confidence/credible interval), ideally using structured tables or plots. | n/a |
| Results of syntheses | 20a | For each synthesis, briefly summarize the characteristics and risk of bias among contributing studies. | Yes, section ‘Methods’, sub-section ‘Methodological Quality of Studies’ |
|  | 20b | Present results of all statistical syntheses conducted. If meta-analysis was done, present for each the summary estimate and its precision (e.g., confidence/credible interval) and measures of statistical heterogeneity. If comparing groups, describe the direction of the effect. | Narrative synthesis of studies presented in Table 3 |
|  | 20c | Present results of all investigations of possible causes of heterogeneity among study results. | n/a |
|  | 20d | Present results of all sensitivity analyses conducted to assess the robustness of the synthesized results. | n/a |
| Reporting biases | 21 | Present assessments of risk of bias due to missing results (arising from reporting biases) for each synthesis assessed. | n/a |
| Certainty of evidence | 22 | Present assessments of certainty (or confidence) in the body of evidence for each outcome assessed. | n/a |
| **DISCUSSION** | | |  |
| Discussion | 23a | Provide a general interpretation of the results in the context of other evidence. | Yes, section ‘Results’, sub-section ‘SMR Characteristics in Resting-state studies’ and ‘SMR Characteristics in Movement-related studies' |
|  | 23b | Discuss any limitations of the evidence included in the review. | Yes, section ‘Discussion’ |
|  | 23c | Discuss any limitations of the review processes used. | Yes, section ‘Discussion’ |
|  | 23d | Discuss implications of the results for practice, policy, and future research. | Yes, section ‘Discussion’ |
| **OTHER INFORMATION** | | |  |
| Registration and protocol | 24a | Provide registration information for the review, including register name and registration number, or state that the review was not registered. | Yes, section ‘Methods’, sub-section ‘Protocol Registration’; protocol registration number provided |
|  | 24b | Indicate where the review protocol can be accessed, or state that a protocol was not prepared. | Yes, section ‘Methods’, sub-section ‘Protocol Registration’; link to access review protocol provided |
|  | 24c | Describe and explain any amendments to information provided at registration or in the protocol. | n/a |
| Support | 25 | Describe sources of financial or non-financial support for the review, and the role of the funders or sponsors in the review. | n/a |
| Competing interests | 26 | Declare any competing interests of review authors. | Yes, Declarations of Interest reported |
| Availability of data, code, and other materials | 27 | Report which of the following are publicly available and where they can be found: template data collection forms; data extracted from included studies; data used for all analyses; analytic code; any other materials used in the review. | n/a |

**Supplementary Table 3.** PRISMA-S Checklist

| **Section/topic** | **Item#** | **Checklist item** | **Location(s) Reported** |
| --- | --- | --- | --- |
| **INFORMATION SOURCES AND METHODS** | | | |
| Database name | 1 | Name each individual database searched, stating the platform for each. | Yes, section ‘Methods’, sub-section ‘Search Strategy and Selection Criteria’, main-text Figure 1, Supplementary Figure 1 |
| Multi-database searching | 2 | If databases were searched simultaneously on a single platform, state the name of the platform, listing all the databases searched. | n/a |
| Study registries | 3 | List any study registries searched. | n/a |
| Online resources and browsing | 4 | Describe any online or print source purposefully searched or browsed (e.g., tables of contents, print conference proceedings, web sites), and how this was done. | n/a |
| Citation searching | 5 | Indicate whether cited references or citing references were examined, and describe any methods used for locating cited/citing references (e.g., browsing reference lists, using a citation index, setting up email alerts for references citing included studies). | Yes, section ‘Methods’, sub-section ‘Search Strategy and Selection Criteria’ |
| Contacts | 6 | Indicate whether additional studies or data were sought by contacting authors, experts, manufacturers, or others. | n/a |
| Other methods | 7 | Describe any additional information sources or search methods used. | Yes, section ‘Methods’, sub-section ‘Search Strategy and Selection Criteria’ |
| **SEARCH STRATEGIES** | | | |
| Full search strategies | 8 | Include the search strategies for each database and information source, copied and pasted exactly as run. | Yes, Supplementary Figure 1 |
| Limits and restrictions | 9 | Specify that no limits were used, or describe any limits or restrictions applied to a search (e.g., date or time period, language, study design) and provide justification for their use. | Yes, section ‘Methods’, sub-section ‘Search Strategy and Selection Criteria’ |
| Search filters | 10 | Indicate whether published search filters were used (as originally designed or modified), and if so, cite the filter(s) used. | Yes, Supplementary Figure 1 |
| Prior work | 11 | Indicate when search strategies from other literature reviews were adapted or reused for a substantive part or all the search, citing the previous review(s). | n/a |
| Updates | 12 | Report the methods used to update the search(es) (e.g., rerunning searches, email alerts). | n/a |
| Dates of searches | 13 | For each search strategy, provide the date when the last search occurred. | Yes, Supplementary Figure 1 |
| **PEER REVIEW** | | | |
| Peer review | 14 | Describe any search peer review process. | n/a |
| **MANAGING RECORDS** | | | |
| Total Records | 15 | Document the total number of records identified from each database and other information sources. | Yes, section ‘Methods’, sub-section ‘Selection of Studies’, main-text Figure 1 |
| Deduplication | 16 | Describe the processes and any software used to deduplicate records from multiple database searches and other information sources. | Yes, main-text Figure 1 |
|  |  |  |  |
